# Do Suicide Attempts of Mood Disorder Patients Directly Increase the Risk for a Reattempt?

**DOI:** 10.3389/fpsyt.2020.547791

**Published:** 2020-11-26

**Authors:** Kari I. Aaltonen, Tom Rosenström, Pekka Jylhä, Irina Holma, Mikael Holma, Sanna Pallaskorpi, Kirsi Riihimäki, Kirsi Suominen, Maria Vuorilehto, Erkki T. Isometsä

**Affiliations:** ^1^Department of Psychiatry, University of Helsinki and Helsinki University Hospital, Helsinki, Finland; ^2^Mental Health Unit, Finnish Institute of Health and Welfare, Helsinki, Finland; ^3^Department of Psychology and Logopedics, University of Helsinki, Helsinki, Finland; ^4^Department of Mental Health and Substance Abuse Services, Department of Health and Social Services, Helsinki, Finland

**Keywords:** major depressive disorder, bipolar disorder, major depressive episode, suicidal act, suicide attempts

## Abstract

**Background:** Preceding suicide attempts strongly predict future suicidal acts. However, whether attempting suicide *per se* increases the risk remains undetermined. We longitudinally investigated among patients with mood disorders whether after a suicide attempt future attempts occur during milder depressive states, indicating a possible lowered threshold for acting.

**Methods:** We used 5-year follow-up data from 581 patients of the Jorvi Bipolar Study, Vantaa Depression Study, and Vantaa Primary Care Depression Study cohorts. Lifetime suicide attempts were investigated at baseline and during the follow-up. At follow-up interviews, life-chart data on the course of the mood disorder were generated and suicide attempts timed. By using individual-level data and multilevel modeling, we investigated at each incident attempt the association between the lifetime ordinal number of the attempt and the major depressive episode (MDE) status (full MDE, partial remission, or remission).

**Results:** A total of 197 suicide attempts occurred among 90 patients, most during MDEs. When the dependencies between observations and individual liabilities were modeled, no association was found between the number of past suicide attempts at the time of each attempt and partial remissions. No association between adjusted inter-suicide attempt times and the number of past attempts emerged during follow-up. No indication for direct risk-increasing effects was found.

**Conclusion:** Among mood disorder patients, repeated suicide attempts do not tend to occur during milder depressive states than in the preceding attempts. Previous suicide attempts may indicate underlying diathesis, future risk being principally set by the course of the disorder itself.

## Introduction

Patients with depressive or bipolar disorder have a high risk for suicidal ideation, suicide attempts (1–3), and death by suicide (4, 5). Suicidal ideation occurs almost without exception with clinically significant depression (6, 7) and resolves with a decline in depressive symptoms (8–10). The differences in quality and intensity of partly overlapping factors influencing the formation of suicidal ideation and then risk for suicide attempts during mood episodes remain poorly understood (11).

Time plays a major role in suicidal behavior in major depressive disorder (MDD) and bipolar disorder (BD) (1). Over 70% of attempts occur during major depressive episodes (MDEs) and few (≤8%) during remission, while mixed states in BD constitute very high-risk periods (7, 12, 13). Although cross-sectional epidemiological surveys and meta-analytic categorical data have suggested that depression mainly influences suicidal ideation or challenges its clinical significance (14, 15), suicide attempts over time do accumulate among patients with the most severe, altering, and persistent symptoms (12, 13, 16).

Current theories on suicidal behavior are based on stress-diathesis models (17). The *stress-diathesis model* by van Heeringen and Mann (18) posits that external stressors, including the exacerbation of psychiatric disturbance, interact with the diathesis of suicidal behavior rooted in neurobiological dysfunctions. The *cognitive-behavioral theory* (CBT) recognizes three main constructs, including dispositional vulnerability factors, cognitive processes associated with psychiatric disturbance, and cognitive processes associated with suicidal acts (19). According to the CBT, transition from suicidal thoughts to action happens when an individual's tolerance for distress becomes overwhelmed. The cognitive processes associated with suicidal acts include suicide schemas that are postulated to strengthen with each suicide attempt, thus becoming more easily triggered in the future. Theoretical considerations also take into account the possibility of past suicide attempts lowering the within-individual threshold of tolerance (20). Three psychological theories termed to form ideation-to-action framework theories seek to explain suicidal thoughts and actions as separate processes. First, the *Interpersonal Theory of Suicide (ITS)* (21) postulates that suicidal desire results from an individual experience of thwarted belongingness and perceived burdensomeness (and related hopelessness). The model then separates the construct of the capability to engage in suicidal acts resulting from both hereditary and acquired components. According to Joiner et al. (22), a non-fatal suicide attempt may represent the most direct factor increasing this capability. Second, the *integrated motivational-volitional (IMV)* model (23) hypothesizes that motivational factors characterized by defeat and entrapment drive emergence of suicidal ideation, whereas acquired capability among other volitional factors contributes to suicide attempts. One of the key premises of the IMV model is that suicidal behavior intensifies suicidal ideation and intent so that over time this may lead to accelerated action. Third, the *Three-Step Theory (3ST)* (24) postulates that the experience of pain and hopelessness drives the emergence of suicidal ideation, which is intensified in the case of low connectedness. According to the 3ST, capacity and progression from ideation to suicide attempts is determined by an array of dispositional and practical factors and acquired capability. This commonly suggested acquired capability for suicide could be associated with a lowered threshold for acting and attempts during milder symptomatic levels, but this association remains to be investigated.

A clear consensus exists of a suicide attempt being a major risk indicator for a subsequent attempt (25, 26). However, the views on which mechanisms give rise to this association are divergent. Briefly, considering that the origins of suicidal behavior are multifactorial (27), a suicide attempt could index the interaction of dispositional and acute state-related factors. More severe and recurrent mood episodes could also predispose reattempts directly or among vulnerable individuals (1). In addition to marking a latent vulnerably factor, an attempt could directly increase the likelihood of a subsequent attempt or denote a mixture of both (28, 29). The stress-diathesis model (18) emphasizes the state and trait model, whereas the ideation-to-action theories (ITS, IMV, and 3ST) propose additional elements of acquired capability directly through past suicide attempts to increase the risks for reattempts. A lowered fear of death and an increased pain tolerance could contribute to this acquired capability (21), and repeatedly activated suicidal cognitions and behaviors could lead to their intense reactivation more readily (20, 23, 29). However, simple behavioral sensitization models hold uncertainties (30). The stress-diathesis model could be dynamic so that increasing capability could lead to a suicide attempt at lower levels of suicidal ideation, or vice versa (31).

The hypothesis of a suicide attempt directly increasing the likelihood of a subsequent suicide attempt has rarely been addressed in longitudinal clinical samples. Previous findings indicate that each past suicide attempt at baseline increases the risk for a next attempt by approximately a third (32–34). These findings include the shortening of the interval between subsequent suicide attempts as the number increases (35), which could also be accounted for by individuals with habitually short mood cycle lengths (36). Among patients with MDD and a non-fatal suicide attempt who eventually die by suicide, the attempts occur throughout successive episodes, whereas among survivors the attempts occur during earlier but not later episodes (37). Overall, data with individual-level repeated measures are necessary for estimating within-individual latent liabilities for attempting suicide. In addition, among patients with mood disorders individual-level data on the course of mood episodes are indispensable when drawing conclusions about the hypothesized effect of a suicide attempt directly increasing the risk of reattempt, for example, through a lowered threshold for acting.

Based on a large well-characterized prospective mood disorder cohort and life-chart methodology, we aim to examine within an individual whether sequential suicide attempts are associated with a lower level of depression severity. In addition, we explore whether an inverse association exists between the cumulative number of suicide attempts and the time between successive suicide attempts.

## Materials and Methods

### Study Setting and Design

Altogether 597 patients within three representative screening-based mood cohorts were followed for 5 years [191 patients with BD in the Jorvi Bipolar Study (JoBS); 269 patients with MDD in the Vantaa Depression Study (VDS); and 137 patients with unipolar depressive disorder in the Vantaa Primary Care Depression Study (PC-VDS)]. The projects were collaborations between the University of Helsinki and Helsinki University Central Hospital, Department of Psychiatry; the Department of Public Health Solutions, Mental Health Unit, National Institute for Health and Welfare, Helsinki, Finland; and the Primary Health Care Organization of the City of Vantaa, Finland. The appropriate ethics committees approved the study design. The detailed methodologies of each cohort from baseline to the 5-year follow-up have been previously published (38–43).

### Screening and Baseline Evaluation

A total of 3,555 primary or secondary care patients showing symptoms of a mood episode were screened for either bipolar disorder (JoBS), MDD (VDS), or unipolar depressive disorder (PC-VDS). Informed consent was requested after full disclosure of the study protocols. The DSM-IV axis I diagnoses were made by structured clinical interview for DSM-IV disorders, SCID-I/P (44) in JoBS and PC-VDS, and by schedules for clinical assessment of neuropsychiatry, SCAN (45) in VDS. The axis II diagnoses were made by structured clinical interview for DSM-IV personality disorders, SCID-II for DSM-IV (46) in JoBS and PC-VDS, and SCID-II for DSM-III-R in VDS. Most interviews were conducted by psychiatrists or psychiatric residents and two skilled clinical psychologists. The interrater agreements (kappa) ranged from 0.86 to 1.00. A detailed overview of the study methods, flow, and ratings is presented in Table 1.

**Table 1:** Materials and methods in the Jorvi bipolar study (JoBS), the Vantaa depression study (VDS), and the Vantaa primary care depression study (PC-VDS).

	**JoBS**	**VDS**	**PC-VDS**
Sampling period	Jan 1, 2002–Feb 28, 2003	Feb 1, 1997–May 31, 1998	Jan 2, 2002–Dec 31, 2002
Setting	Department of Psychiatry, Jorvi Hospital, Helsinki University Central Hospital, Espoo, Finland (catchment area 261,116 in 2002)	Department of Psychiatry of Peijas Medical Care District, Helsinki University Central Hospital, Vantaa, Finland (catchment area 169,000 in 1997)	Primary Healthcare Organization of the City of Vantaa, Finland Three health centers and two maternity clinics serving two districts in the city of Vantaa on population basis (catchment area 63,400 in 2002).
Screening	All psychiatric in- and outpatients aged 18–59 years Seeking treatment Referred to treatment In an acute deteriorating clinical state among patients within secondary care	All psychiatric in- and outpatients aged 20–59 years Seeking treatment Referred to treatment In an acute deteriorating clinical state among patients within secondary care	Consecutive primary care patients aged 20–69 years in general practitioners' waiting room on randomly selected days, stratified for day of the week and month, and time of year.
Screening procedure	(1) Mood disorders questionnaire, 7/13 items positive, or (2) Clinical suspicion of BD (*n* = 28)	(1) One of five screening questions for depression from SCAN, or (2) Scale for suicide ideation, score ≥6	In two phases: (1) PRIME-MD: a positive item for depressive mood or anhedonia during the last month, and (2) telephone interview: confirmed presence of one main symptom of DSM-IV MDD (according to SCID-I/P)
Excluded in screening	ICD-10 schizophrenia	ICD-10 schizophrenia, or BD-I	Current psychiatric secondary care contact, primary psychotic disorder, bipolar and organic mood disorders, alcohol use disorders preventing 2 weeks' abstinence for interview, insufficient communication or Finnish language skills, poor general health status or medical emergency preventing screening
Total screened	1,630	806	1,119
Screened positive	546	703	402 (PRIME-MD), 375 telephone interview
Coverage of screening	46 (2.8%) declined from screening 49 (9.0%) with a positive screen declined from interview 7 not contacted	161 (22.9%) with a positive screen declined from an interview	8 declined from PRIME-MD screening 27 (6.7%) declined from telephone interview 10 (2.5%) declined from diagnostic interview
Interviewed	490 (SCID-I/P)	542 (SCAN)	175 (SCID-I/P)
Inclusion criteria	BD type I or II with a new DSM-IV depressive, manic, hypomanic, mixed, or depressive mixed episode.	DSM-IV MDD with a new depressive episode	DSM-IV MDD, dysthymia, or partial depression (two to four symptoms) with or without history of lifetime MDD
Eligible	201 (10 declined)	269	140 (3 declined)
Cohort	191 (65 inpatients and 126 outpatients) BD I: 90 BD II: 101 Men: 90 Women: 101 Age (mean) at baseline: 37.7 years (SD 12.2)	269 (46 inpatients, 223 outpatients) Men: 73 Women: 196 Age (mean) at baseline: 39.6 years (SD 11.1)	137 outpatients from primary care Current MDD: 91 Partial MDD: 46 (includes four patients with dysthymia) Men: 33 Women: 104 Age (mean) at baseline: 45.3 years
Patients at 6-month follow-up	176 (92.1%)	229 (85.1%)	–
Patients at 18-month follow-up	160 (83.8%)	207 (76.9%)	127 (93%)
5-year follow-up	113 (61.7%)	182 (67.7%)	112 (82.0%)
Number of patients Switch of diagnosis Participants vs. non-participants Follow-up time	1 schizoaffective disorder No difference in prevalence of suicide attempts before or during index episode at baseline. Median 62.2 months	29 BD, 1 schizophrenia, 2 schizoaffective disorder No difference in prevalence of suicide attempts or suicidal ideation before or during index episode at baseline. Median 62.4 months	6 BD No difference in age, gender, or baseline depression severity. Median 62.9 months
Diagnostic reliability at baseline	20 random videotaped diagnostic interviews; kappa coefficient for BD = 1.0	20 videotaped diagnostic interviews; Kappa coefficient for MDD = 0.86 (95% CI = 0.58–1.00)	20 random videotaped diagnostic interviews; kappa coefficient for current full and partial MDD = 1.0
Symptom assessment	BAI, BDI, BHS, HAM-D, SSI, YMRS (baseline and at all follow-ups)	BAI, BDI, BHS, HAM-D, SSI (baseline and at all follow-ups), in addition BDI monthly for first 6 months	BAI, BDI, BHS (baseline, 3, 6, and 18 months, and 5 years), and HAM-D, SSI (baseline, 18 months, and 5 years)

*BAI, Beck anxiety inventory; BD, bipolar disorder; BDI, Beck depression inventory; BHS, Beck hopelessness scale; DSM-IV, Diagnostic and Statistical Manual of Mental Disorders, 4^th^ edition; HAM-D, Hamilton rating scale for depression; ICD-10, International Classification of Disease, 10^th^ edition; JoBS, Jorvi bipolar study; MDD, major depressive disorder; PC-VDS, Vantaa primary care depression study; PRIME-MD, primary care evaluation of mental disorders; SCAN, schedules for clinical assessment of neuropsychiatry; SCID-I/P, structured clinical interview for DSM-IV disorders; SSI, the scale for suicidal ideation; VDS, Vantaa depression study; YMRS, young mania rating scale*.

### Follow-Up and Life-Chart Methodology

Patients were re-interviewed at 6, 18 months, and 5 years in JoBS (SCID-I/P) and VDS (SCAN 2.0 at 6 and 18 months and SCID-I/P at 5 years), and at 18 months and 5 years in PCD-VDS (SCID-I/P). In addition, patients were extensively assessed with rating scales on clinical symptoms and suicidal ideation. A total of 407 patients (68.2%) were interviewed at the 5-year follow-up. Detailed information on the study design and follow-up procedures is presented in Table 1.

A graphic life-chart was created at all interviews based on DSM-IV criteria to depict the course of the mood disorder throughout the follow-up. This graphic life-chart was created by combining information from all available sources including the diagnostic interviews, study rating scales, and medical and psychiatric records. In addition, information from scales used in routine clinical practice was variably available. During these extensive interviews, a judgement was made of the course of the mood disorder during the follow-up. Changes in mood episodes or phases were timed both by combining all aforementioned information and by using probes of important life events to enhance the accuracy of the chart. The phases of depressive symptoms were classified based on DSM-IV criteria as (1) remission (no MDE criteria symptoms), (2) partial remission (one to four of the nine symptoms), and (3) full MDE (at least five criteria symptoms). In JoBS, in addition hypomanic, manic, mixed, mixed depressive, cyclothymic, or substance-induced phases and subsyndromal states of hypomania of BD were recorded. However, the minimum duration for hypomania was two instead of 4 days, and mixed depressive states were included.

### Suicidal Behavior

A suicide attempt, by definition, was specified as self-injurious behavior involving at least some degree of intention to die (thus excluding self-harm without suicidal intention). At baseline, we examined the lifetime number of suicide attempts by combining the information from both interviews and psychiatric records. At follow-ups, the occurrence of suicide attempts was investigated by patient interviews and psychiatric and medical records. Mood episodes and their timing were assessed first and independently from suicide attempts to limit circumventing the evaluation of a mood state to be based on suicidal behavior, and vice versa.

### Statistical Methods

#### Association Between the Number of Previous Suicide Attempts and MDE Status

We investigated the association between the number of previous suicide attempts and clinical MDE status at the time of a new monitored attempt. The number of previous suicide attempts at the time of an attempt formed a count variable, which can be modeled using Poisson regression (47). Note that lifetime suicide attempts prior to baseline were determined using medical records and interviews. The dependencies between observations (i.e., sequential suicide attempts of an individual during follow-up) were controlled using multilevel (a.k.a. random-effect) modeling as implemented in the “lme4” R package, version 1.1-12 in the R software, version 3.3.2 (47–49).

Specifically, the multilevel Poisson regression model assumes that the logarithm of the expected number of suicide attempts (Y) preceding the current one for patient *i* at attempt *j* is

(1) $$\begin{array}{r} \log\left(E\left[Y_{ij}|x_{ij},f_{ij},\eta_{i}\right]\right)=\beta_{0}+\beta_{1}x_{ij}+\beta_{2}f_{ij}+\eta_{i}, \end{array}$$

where β_0_ is the baseline rate (those in full remission), *x*_*ij*_ is a dummy indicator for partial remission, and β_1_ is the associated effect difference to full remission, *f*_*ij*_ is a dummy indicator for full MDE and β_2_ is its associated effect difference to full remission, and η_*i*_ is the patient-specific liability of patient *i*. If many preceding suicide attempts at the time of a new attempt are associated with higher proportions of partial remission and remission stages, negative β_1_ and β_2_ coefficients are expected. The null hypothesis is β_1_ = β_2_ = 0. Substantive interpretation of the random intercept (*η*_*i*_) is complicated by the fact that it reflects both the deterministic condition that a second suicide attempt during the follow-up is preceded by *n* + 1 attempts if the first attempt was preceded by *n* attempts (etc.), and the stochastic condition of the overall higher *n* values in those with repeated attempts.

#### Statistical Power

We studied our power to detect different effect sizes using a simulation procedure typical in multilevel modeling (47). Poisson-distributed observations were generated with an expectation equal to our empirically observed rate of previous suicide attempts at the time of a new attempt (4.35 on average, s.d. = 4.97, median = 3). Altogether 3,000 datasets equal in size to our sample (number of individuals and their repeated observations) were simulated for each studied effect size and level of random-effect variance. The proportion of significant tests among the 3,000 datasets characterizes the statistical power per effect size and random-effect variance.

#### Testing Acceleration of Suicidal Behavior

When studying cycle length acceleration in MDEs, over-representation of highly and consistently recurrent individuals among those with short inter-morbid intervals could result in the appearance of shortening of intervals between MDEs (36). To assess whether inter-morbid intervals truly declined as a function of the number of previous MDEs, Anderson et al. (36) subtracted within-individual averages from patients' inter-morbid intervals before investigating the association between inter-morbid interval and the cumulative number of MDE—lest the findings be confounded by stable individual differences in recurrence rates. In analogy, we took inter-suicide attempt intervals for patients with >2 attempts during follow-up (*n* = 22, having 99 repeat attempts), subtracted within-patient averages from time intervals of successive attempts, and investigated the association between the cumulative number of repeated suicide attempts and the time since a previous attempt. We estimated both ordinary correlation coefficients and a linear mixed model with patient-specific random intercepts, plus scatterplots.

## Results

**Figure 1:**
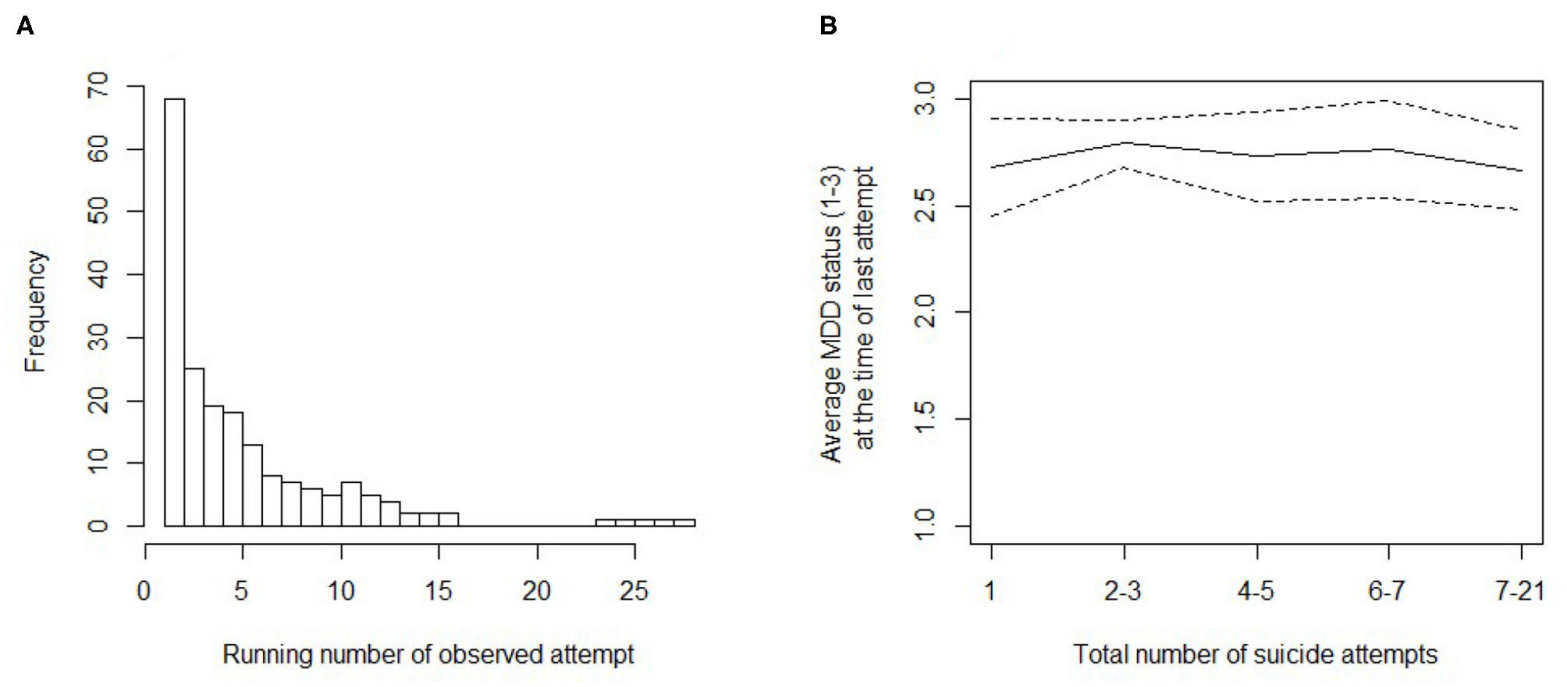
Number of suicide attempts and status of major depressive episode (MDE). **(A)** Histogram of the lifetime running number of the attempt for each suicide attempt observed during the follow-up. **(B)** Average status of MDE (values: 1 = in remission, 2 = partial remission, and 3 = full MDE) at the time of the last attempt in patients with a different lifetime number of suicide attempts. The dashed lines give 95% Wald confidence intervals for the average (solid line).

Of the 597 patients included at the baseline, individuals with a suicide attempt during a mixed-state MDE (*n* = 15) or medication-induced hypomania (*n* = 1) during the follow-up were excluded. Of the remaining 581 patients included here, altogether 90 had a total of 197 suicide attempts during the follow-up (altogether 75 attempts among patients with BD, and 122 attempts among patients with unipolar depression). These attempts represent our sample of interest in this study. Table 2 and Figure 1A describe the distribution of the lifetime cumulative number of attempts during follow-up (attempts preceding baseline included).

**Table 2:** Distribution of lifetime cumulative number of attempts during follow-up^†^.

**Cumulative number of attempts**	**Average MDD status**	**s.d. of MDD status**	**Group size**
1	2.68	0.65	31
2–3	2.79	0.45	62
4–5	2.73	0.65	37
6–7	2.76	0.54	21
7–21	2.67	0.64	45

*MDD values: 1 = remission, 2 = partial remission, and 3 = full MDE*.

† *includes attempts preceding baseline*.

### Is a Greater Cumulative Number of Past Suicide Attempts Associated With Successively Less Severe Depression at the Time of New Suicide Attempt?

In terms of an average MDE status (remission, partial remission, and full MDE), the data showed no clear association between the within-individual cumulative number of suicide attempts and the clinical status of depression at the time of each attempt. Instead, most attempts occurred in full MDE (Table 2 and Figure 1B). An ordinary analysis of variance would support this conclusion [*F*_(4,191)_ = 0.382, *p* = 0.821], but is subjected to bias due to dependent observations and the possible dependence of MDE status on time since the MDD-based recruitment to study (baseline). The latter remains an unlikely confounder because the time from baseline to an attempt and MDE status at the time of an attempt were uncorrelated (polyserial *r* = −0.006). To model observation dependencies (trait-like differences in liability), we implemented a multilevel Poisson model predicting the number of previous suicide attempts at the time of a new attempt with (dummy) indicators of MDE status. Statistical power of the approach was first investigated.

#### Statistical Power in Multilevel Modeling

**Figure 2:**
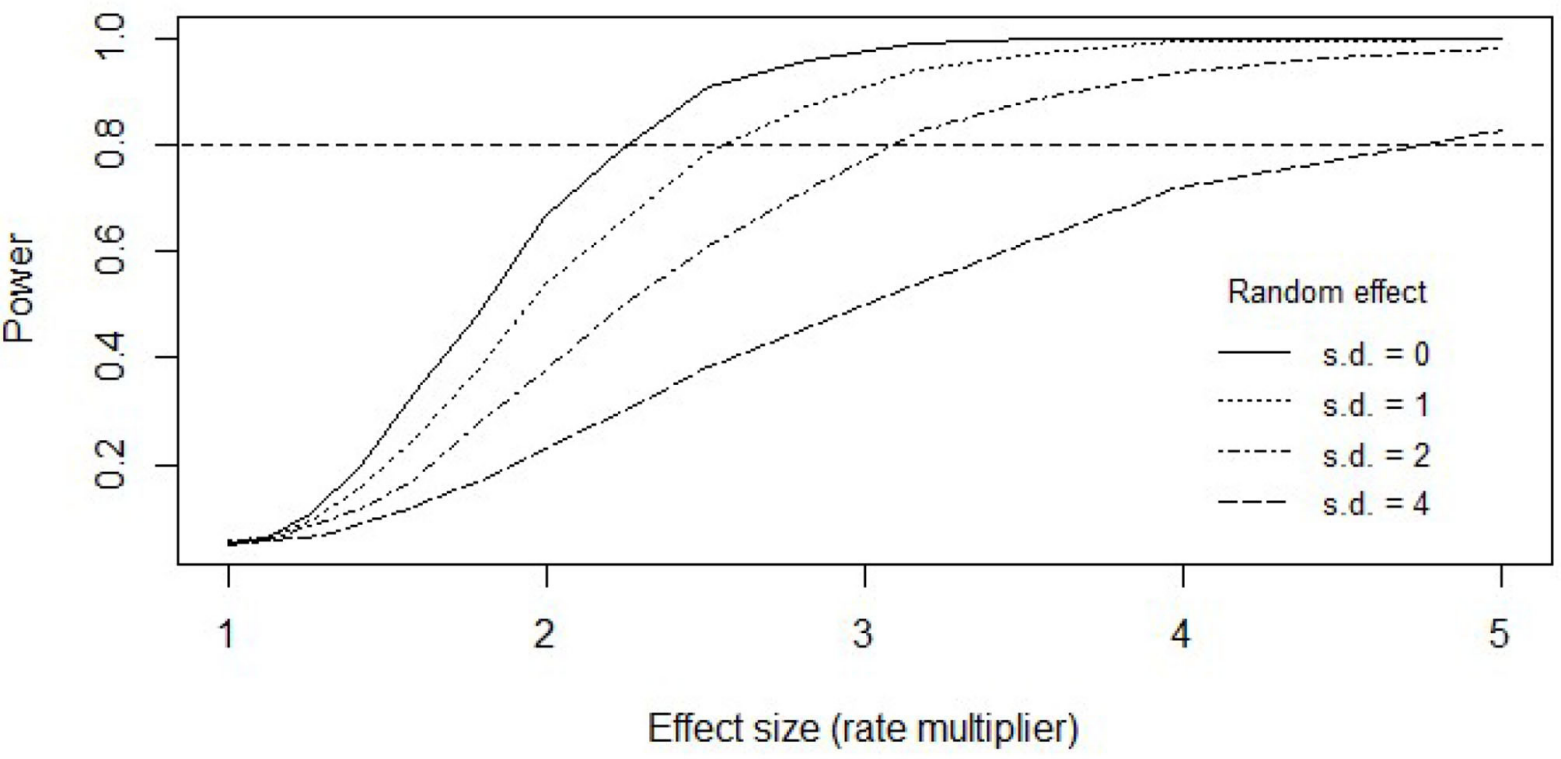
Statistical power in the Poisson multilevel model test for the association between the status of major depressive episode (MDE) and the number of previous attempts at the time of a new attempt. As the effect of MDE status on suicide attempts increases (*x*-axis), chances to detect it increase too (statistical power; *y-*axis). The less the between-patient differences explain differences in suicidality, the more rapid the increase in our ability to detect an effect of MDE status. That is, power is a function of both attempt multiplier per depression-status difference (effect size) and between-individual differences in the overall numbers of suicide attempts (random effect standard deviance; greater the value greater the differences). Statuses of a major depressive episode can be “remission” (reference category), “partial remission,” or “full MDE.” Horizontal dashed line highlights the point of 80% chance to detect an existing omnibus effect in data like ours.

Figure 2 shows the statistical power (probability with repeated sampling) to detect an existing effect in the present sample, assuming a one-step lower, or less severe, MDE status at the time of an attempt is associated with a rate ratio (RR)-fold increase in the number of preceding suicide attempts. That is, the RR is the effect size. Statistical power was studied for four separate cases, assuming different amounts of individual differences in liabilities for attempting suicide. Normally distributed liabilities (*η*_*i*_ in Equation 1) with standard deviations 0, 1, 2, and 4 were tested, corresponding to RRs of 1, 2.7, 7.4, and 54.6, respectively. The RR value of 54.6 represents a case of large between-individual differences in liability. With the smaller between-individual differences, we had a good power to detect 3-fold numbers of suicide attempts associated with a one-step decrease in MDE status; with large between-individual differences, only strong (5-fold) effects could be reliably detected. However, model estimates indicated that the data were in the smaller range of between-individual differences (i.e., σ ≈ 1 in Table 3), suggesting reasonable statistical power for our analysis.

**Table 3:** Multilevel Poisson regression predicting the number of previous suicide attempts at the time of new attempt with MDE status (fixed effects) and individual-specific liabilities (random effect).

**Effect type**	**Variable**	**β**	**RR**	**CI**	***p*-value**
Fixed	Intercept	0.536	1.708	1.08–2.63	0.018
Fixed	Partial remission	−0.191	0.826	0.58–1.18	0.283
Fixed	Full MDE	0.010	1.010	0.70–1.48	0.957
		**σ***_**η**_*	**CI**		
Random	Intercept	1.060	0.85–1.33		

*β = regression coefficient, σ = random-effect (trait, or **η** in Equation 1) variance*.

*MDE, major depressive episode; RR, implied rate ratio/multiplier; CI, confidence interval of left-hand value*.

#### Real-Data Estimates in the Multilevel Model

Table 3 shows results from a multilevel Poisson regression model in predicting the number of previous suicide attempts at the time of a new attempt using MDE status and a random intercept as an independent variable. We found no evidence for dependence between the number of previous suicide attempts and MDE status at the time of a new attempt [Table 3; $\chi_{\left(2\right)}^{2}$ = 2.62, *p* = 0.270 for likelihood-ratio test of difference between models with and without MDE status indicators, as in the power simulations]. Adjusting for fixed and random effects of time between the current attempt and the study baseline [$\chi_{\left(2\right)}^{2}$ = 1.40, *p* = 0.496] did not change the situation.

### Does the First Lifetime Suicide Attempt Occur in More Severe MDE Than Repeated Attempts?

Altogether 31 first attempts occurred during the follow-up period. Contrary to the general supposition, on average first attempts occurred in less severe MDEs (average MDE status = 2.68, s.d. = 0.65) than repeat attempts (average MDE status = 2.74, s.d. = 0.56). By omitting observation dependencies, the difference (Cohen's *d* = 0.11) was statistically non-significant (Welch two-sample *t* = −0.495, *d.f*. = 38.81, *p* = 0.623). When considering observation dependencies by logistic multilevel regression, MDE status was still unassociated with an observed suicide attempt being either first or next in order [$\chi_{\left(2\right)}^{2}$ = 1.16, *p* = 0.559].

### Do Time Intervals Between Suicide Attempts Shorten as the Number of Attempts Grows?

**Figure 3:**
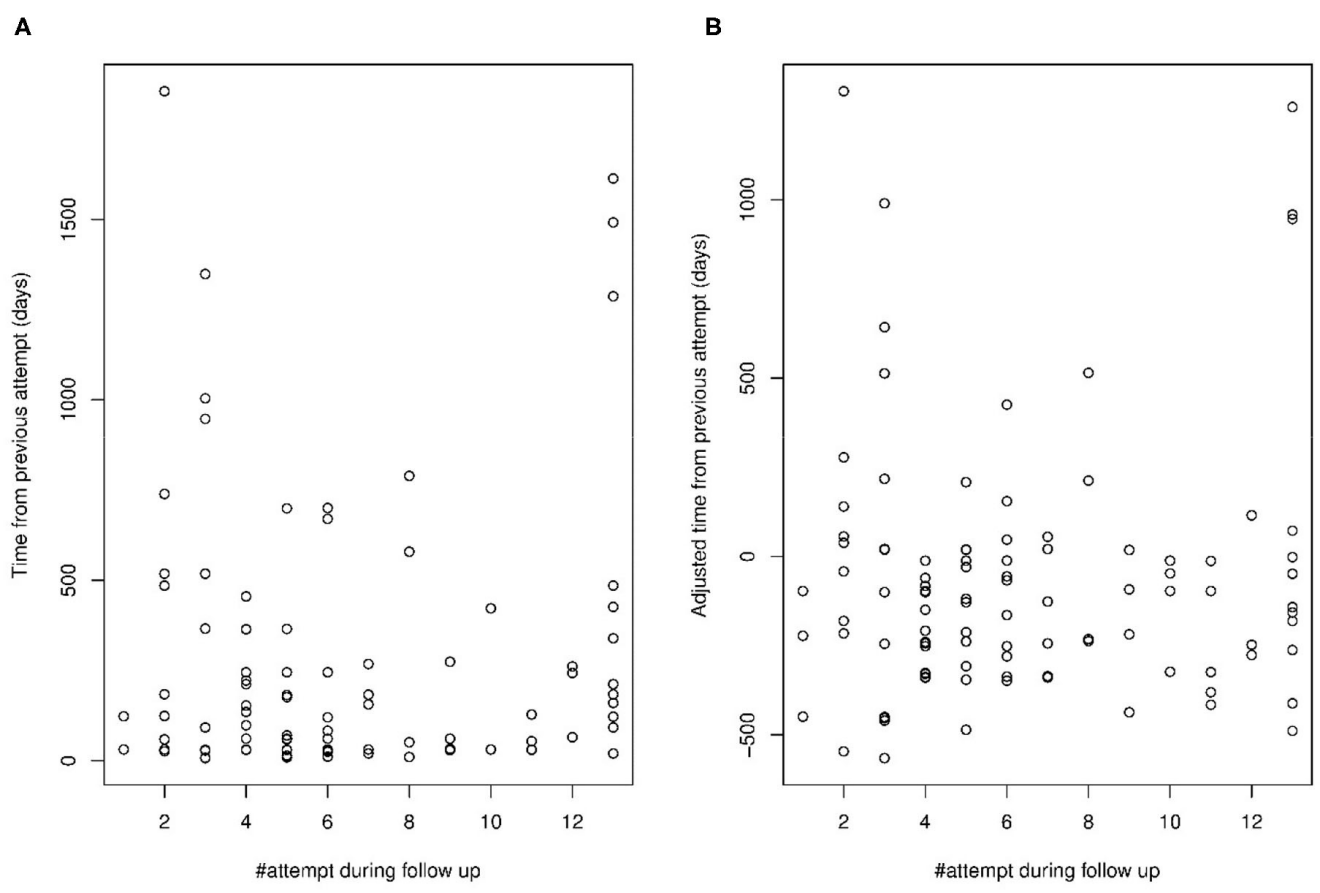
Time intervals between suicide attempt during the follow-up per attempt number **(A)** unadjusted for within-patient average intervals, and **(B)** adjusted for within-patient average intervals.

The above analyses argued against the idea that the risk of a new suicide attempt grows with each attempt because repeat attempts occur in progressively less severe depression. The risk of repeating a suicide attempt could still grow by each attempt because depression or other triggers of suicidal behavior occur more often, in accelerating rapidity. Considering the unadjusted time intervals between successive suicide attempts, it may seem like the interval lengths are negatively associated with the cumulative number of repeat attempts during the follow-up (Figure 3A). However, adjusting for within-patient average intervals abolishes this impression (Figure 3B), suggesting it merely occurred because the patients with many suicide attempts had an overall higher rate of attempts than others, not because they underwent rate acceleration by attempt. Neither the correlation (*r* = 0.06, *p* = 0.537) nor fixed effect of a linear mixed model (b = 6.04, s.e. = 9.76, *p* = 0.532) was significant when looking for an association between attempt number and time between attempts. Although repeat attempts beyond 13 were lumped together in these data, their removal did not influence the conclusion (*r* = −0.13, *p* = 0.224, *n* = 87; b = −13.74, s.e. = 11.21, *p* = 0.216).

## Discussion

We used longitudinal one-of-a-kind individual-level data to investigate the widely held hypothesis that a suicide attempt directly increasing the risk for a reattempt. Specifically, we prospectively and by individual-level analyses examined whether successive suicide attempts occurred among patients with MDD or BD at increasingly lower levels of depression. The influence of the course and severity of depressive morbidity was considered using life-chart data. The main finding, with both theoretical and clinical implications, is that the data provided no evidence for a suicide attempt directly increasing the risk for a reattempt.

To our understanding, the study hypothesis was tested in a unique study design. The major strength was the availability of the longitudinal data on the course of the mood episodes by life-chart methodology, combined with the accurate timing of suicide attempts. Individual-level data enabled the modeling of within-individual differences in clinical status at the time of each sequentially monitored suicide attempt. The screening-based sampling yielded previously undiagnosed patients, assuring representativeness. Pooling data from the three representative cohorts to increase patient numbers was possible due to similar study methodologies. The study diagnostics were based on semi-structured interviews (SCID-I/P; SCAN) with high inter-rater agreement. The drop-out rate during long-term follow-up was relatively limited and only a few patients missed all follow-ups. Although we could have had a better statistical power for small effect sizes, the power was still good for moderate effect sizes. Data of this kind are rare, and we did not observe any indication that the null hypothesis should be rejected.

Although at the follow-ups, extensive information was gathered by interviews and scales, the main limitation of the study is the depiction of life-charts by three-step ordinary scales of remission, partial remission, and full MDE. This restriction may have resulted in ceiling effects when severity above the diagnostic threshold at each attempt was neither measured nor modeled. However, this is a likely limitation for all prospective clinical studies until more detailed data on a daily basis become available, for example, through ecological momentary assessments. The intensity of suicidal ideation may have varied at the time of the attempt. Ambiguities remain pertinent to underlying the specific constructs of psychological theories of suicidal behavior, which remained unassessed with actual instruments. However, suicidal ideation was, by definition, present at the time of each attempt irrespective of possible theoretical constructs driving its formation. The constructs involving engaging in suicidal acts were implicitly modeled by examining the probability to attempt suicide one more time either at lower levels of depression, or at an accelerating rate from the previous attempt. Both outcomes are clearly defined and are of clinical and theoretical interest. Some circularity between the timing of suicide attempts and the course and severity of MDEs may be unavoidable. To minimize confounding, the life-charts were reconstructed first and independently from the timing of suicide attempts, and information on both was complemented with medical records. In addition, attempting suicide seldom determines whether the diagnostic criteria for an MDE are met (6). Recall biases over the last 3.5-year gap preceding the last follow-up may have resulted in some inaccuracies. Treatment received by the patients was as usual. About 42–78% received adequate treatment in the acute phase, whereas the adequacy of maintenance treatment was compromised as the follow-up progressed (50–54). Treatment received by a patient may have reduced the overall risk of attempting suicide, but to influence testing our study hypothesis, this should have had a direct moderating impact on the relationship between the level of depression severity and threshold for re-attempting suicide in a consecutive series of attempts. It is noteworthy that the findings pertain strictly to patients with depressive or bipolar disorder and may not be otherwise generalized. Lastly, in a sample of repeat attempters, the possibility of selection bias for low-lethality attempters exists and the interpretations for suicide deaths must be made carefully.

Patients with depressive or bipolar disorder are a high-risk group for suicidal behavior, accounting for over half of all suicides (55) and admissions for self-harm (56). Risk for suicide death is highest among those who have previously attempted suicide (57, 58). Clarifying the interrelations between mood episodes, suicidal ideation, and prior suicide attempts as risk factors for repeating acts is important. In this study, longitudinally monitored suicide attempts of an individual occurred with the same likelihood during an MDE as previous attempts. Conversely stated, a reattempt after a suicide attempt was not more likely to occur during partial symptoms of depression, which would have been expected if a suicide attempt directly increases the risk for repetition at lower levels of distress. Neither did the data show a shortening of the inter-attempt interval as the number of suicide attempts increased, a result in line with similar null findings considering inter-episode intervals in MDEs (36). Those patients with a high number of suicide attempts had an overall high frequency of suicide attempts instead of rate acceleration. The findings of this study providing longitudinal and individual-level information agree with others (16, 59) on the importance of temporal and state-dependent understanding of suicidal behavior. Patients with depression and a history of a suicide attempt are at high risk for suicide (58) and form an important target group for suicide prevention (27, 60). Our findings are in alignment with this, but suggest that instead of direct (that is causal) effects, suicide attempts may index other more determinative factors such as more a severe course or characteristics of depression, or other individual factors belonging to the diathesis. While future studies will be needed to clarify the relationship, these may provide important targets for treatment and prevention.

Multifactorial state and trait-related factors, having origins both distally and proximally in time, contribute to suicidal ideation and attempts (27). Theoretically and congruent with stress-diathesis models of suicidal behavior, the data could be interpreted so that a past suicide attempt indicates multifactorial underlying vulnerability as a part of this diathesis. If so, predisposition to attempting suicide could be relatively stable, whereas suicidal ideation and intent vary temporally over the course of depressive episodes. The continuous interaction of these factors in time would then affect the probability for a suicide attempt. According to the interpersonal theory of suicide, acquired capability might also be formed through painful and provocative experiences other than suicide attempts such as childhood maltreatment and exposure to other suicidal behavior. After being formed, this capability could remain relatively stable over time (21). In this cohort, these acquired elements may have actuated *prior to the first attempt* and remained thereafter. Accordingly, childhood adverse experiences may be associated with specific current and definable clinical characteristics, such as borderline personality disorder traits, which may mediate their effects on predisposition to attempting suicide (61). Reduced distress tolerance, including emotional dysregulation and impaired impulse control, overall, contribute to the probability of acting on one's suicidal thoughts (27). Overall, hopelessness and depression contribute to suicidal ideation, whereas impaired self-control including cluster B and impulsive-aggressive traits to risk on acting on one's thoughts (11, 62, 63). However, the symptoms of these factors, commonly viewed as trait-like, actually show to a significant degree intensification along with concurrent depressive symptoms (16, 64–66). Whether diathesis of suicidal behavior could distally unfold during development or strengthen via acquired capability more proximally before the first attempt is open to future research. However, the data here provide no support for the direct within-individual influence of suicide attempts increasing the likelihood for suicide attempts during states below full MDEs. Differentiating and clarifying psychological and clinical characteristics operating at the time of a full mood episode for short-term risk should be an important aim for the field, albeit difficult to study.

To conclude, repetition of a suicide attempt may occur among vulnerable individuals with a concomitant, severe, and chronic course of a mood disorder. This study presented no convincing evidence that a suicide attempt could directly lower the within-individual threshold for a subsequent suicide attempt. While further research is warranted, these findings are informative both clinically and theoretically.

## Data Availability Statement

The datasets presented in this article are not readily available because dataset not publicly available due to restrictions imposed by the Finnish law and research permits. Requests to access the datasets should be directed to erkki.isometsa@hus.fi.

## Ethics Statement

The studies involving human participants were reviewed and approved by Ethical Committee of Helsinki and Uusimaa Hospital District (HUS). The patients/participants provided their written informed consent to participate in this study.

## Author Contributions

KA reviewed the literature, drafted the initial version, and prepared the manuscript. IH, MH, PJ, SP, KR, KS, and MV were active researchers in the respective study cohorts. TR provided statistical expertise and conducted the analyses. EI created the project as the principal investigator. All authors participated in the study concept and design, critical interpretation of data, revisions of the paper, and approval of the final version for submission.

## Conflict of Interest

The authors declare that the research was conducted in the absence of any commercial or financial relationships that could be construed as a potential conflict of interest.
